# Hourly and accurate severe sepsis classification using kernel density estimates

**DOI:** 10.1186/cc12966

**Published:** 2013-11-05

**Authors:** Jacquelyn D Parente, Geoffrey M Shaw, Dominic S Lee, J Geoffrey Chase

**Affiliations:** 1 Centre for Bioengineering, Department of Mechanical Engineering, University of Canterbury, Christchurch, New Zealand; 2 Department of Intensive Care, Christchurch Hospital, Christchurch, New Zealand; 3 Department of Mathematics and Statistics, University of Canterbury, Christchurch, New Zealand

## Background

Sepsis score classifications increase conditionally with concurrent systemic inflammatory response syndrome (SIRS) score, Sequential Organ Failure Assessment score, and clinical intervention. However, hierarchical criteria fail to accurately classify sepsis when related physiological manifestations are resolved, while the underlying infection remains.

## Materials and methods

To enable hour-to-hour sepsis classification, we examined the diagnostic performance of a continuous sepsis score. We identified 36 adult patients in the Christchurch Hospital ICU with sepsis from a patient database. A severe sepsis biomarker was developed from model-based insulin sensitivity, temperature, heart rate, respiratory rate, blood pressures, and SIRS score. Sepsis and nonsepsis patient-hours were categorized by the ACCP/SCCM guidelines, where each category was scored independently, rather than hierarchically. Kernel density estimates were used to classify severe sepsis (including septic shock) of 1,690 hours over 6,550 total hours. Optimal diagnostic performance from the receiver operating characteristic (ROC) curve was determined for in-sample, out-of-sample, and overall estimates.

## Results

The severe sepsis biomarker achieved 86% sensitivity (81 to 94%), 85% specificity (80 to 95%), 0.93 (0.88 to 0.99) area under the ROC curve, 8.2 (4.0 to 19.0) positive likelihood ratio, 0.17 (0.06 to 0.23) negative likelihood ratio, 68% (58 to 87%) positive predictive value, 94% (92 to 98%) negative predictive value, and a diagnostic odds ratio of 116 (17 to 308) at an optimal probability cutoff value of 0.25.

## Conclusions

This clinical biomarker can thus be readily assessed at the bedside to yield a non-invasive and continuous estimate of the probability of severe sepsis. The results show high accuracy as a potential severe sepsis diagnostic and monitoring response to sepsis interventions in real time.

